# Mysterious Sight

**Published:** 1888-05

**Authors:** 


### MYSTERIOUS SIGHT.

Lizzie Zink, ten years of age, is a pupil attending the public school in Union Square district, in Lancaster county, Pa. She lives with Moses Ober, a well-known farmer. The child is subject to periods of apparent sleep, and her condition during these periods is so remarkable as to excite the wonder of the neighborhood in which she lives. Of late, under the slightest excitement, she has been subject to peculiar conditions of semi-consciousness which the neighbors pronounce to be a trance.

At these times Lizzie possesses wonderful mind power. Her reasoning force apparently never leaves her, but, on the contrary, becomes stronger. While at school, a short time ago, the teacher, S. E. Weitzel, of Seville, Perry county, Pa., discovered her to be in a comatose state. When she came into the school building she told a playmate that an old woman had been following her and was coming in through the keyhole. The child, with her eyes tightly closed cried loudly: "Gey weck," the Pennsylvania Dutch for "go away." When requested to open her eyes she said the old women was holding them shut with her fingers.

Teacher Weitzel, becoming alarmed, sent for Farmer Ober, and a number of neighbors hearing of the unusual condition of the girl went to the schoolroom and began an investigation of her state. Figures were packed on the blackboard, and, notwithstanding the fact that the girl's eyes were closed, she readily named all of them correctly. With the same precision she repeated words which were written upon the blackboard by the teacher. All questions were anwered without hesitancy. Letters and figures were written on the floor with chalk at different points, and, to the utter amazement of those present the girl told in every instance both their location and character. The water can, basin, bucket and the wearing apparel of other children in the school were in turn held above her head and behind her, and in every instance the nature of the article was pronounced without hesitancy. When a picture of the school building containing a group of the scholars was placed before Lizzie, she immediately told what it was, and even went so far as to place her finger upon the figures of the teacher and a number of her favorite playmates who appeared in the group.

When Farmer Ober made his appearance in response to a message that had been sent to him, without a word or a signal of any kind announcing his coming, a bright smile spread over the girl's face and her whole appearance changed. She cried out joyfully: "Oh, Mr. Ober, I am so glad you have come." When Lizzie's eyelids were raised it was found that the pupils were turned up in her head and nothing showed except the white portion of the eyeballs. The child was taken home, and remained in the same state for nearly six hours, during which time hundreds of people went to see her.

Many superstitious people are inclined to believe that Lizzie has been bewitched. She has been similarly affected upon a number of previous occasions. After the trance she has no recollection of what has occurred, and it is some time before her mental faculties resume their normal condition.

